# Measuring Activity Performance of Older Adults Using the activPAL: A Rapid Review

**DOI:** 10.3390/healthcare5040094

**Published:** 2017-12-13

**Authors:** Charice S. Chan, Susan E. Slaughter, C. Allyson Jones, Carla Ickert, Adrian S. Wagg

**Affiliations:** 1 Faculty of Agricultural, Life and Environmental Sciences, 2-06 Agriculture Forestry Centre, University of Alberta, Edmonton, AB T6G 2P5, Canada; charice@ualberta.ca; 2 Faculty of Nursing, Edmonton Clinic Health Academy, University of Alberta, Edmonton, AB T6G 1C9, Canada; cickert@ualberta.ca; 3 Faculty of Rehabilitation Medicine, University of Alberta, 8205 114 Street, 3-44C Corbett Hall, Edmonton, AB T6G 2G4, Canada; cajones@ualberta.ca; 4 Department of Medicine, University of Alberta, 1-198 Clinical Sciences Building, 11350-83 Avenue, Edmonton, AB T6G 2P3, Canada; adrian.wagg@ualberta.ca

**Keywords:** accelerometry, physical activity, geriatrics

## Abstract

Current measures of physical activity and sedentary behaviors such as questionnaires and functional assessments are insufficient to provide comprehensive data on older adults. In response, the use of activity monitors has increased. The purpose of this review was to summarize and assess the quality of observational literature on activity measuring of older adults using the activPAL activity monitor. Seventeen databases and a bibliography, compiled by the activPAL creators, were searched. Articles were included if they were in English, were peer-reviewed, included people 65 years or older, measured activity using the activPAL and reported at least one of the following outcomes: step count, hours upright, hours sitting/lying, hours stepping, or hours standing. The search revealed 404 titles; after exclusions 24 were included in the final review. Of these studies, one examined older adults from residential aged care, six from hospital in-patient clinics, nine from outpatient clinics and eight examined community-dwellers. Mean age ranged from 66.0 to 84.2 years. Not all studies reported similar outcome variables, preventing data pooling. The review found a lack of high quality articles. There may be limitations to using the activPAL among older adults but further research is required to examine its use in this population.

## 1. Introduction

According to the World Health Organization, increased physical activity in older adults is associated with lower rates of chronic disease, better cognitive function, healthier body composition, greater bone health, greater levels of functional independence and lower risk of falling [1]. Further, reducing sedentary behaviors, such as long periods of sitting or lying down, may improve metabolic health [2]. Maintaining or increasing physical activity is often a primary aim of clinical rehabilitation for older adults [3]. To assess and address their rehabilitation needs, accurate measurement of the quantity and quality of physical activity is essential [4].

Often, clinicians or researchers use self-report questionnaires or administer functional tests to monitor physical activity and sedentary behavior [3,5]. Several studies have cautioned against the use of questionnaires, as they tend to overestimate physical activity or exclude activity that is completed during activities of daily living [3,6,7] and underestimate time spent in sedentary activities such as watching television [5]. Questionnaires may also be culture- or age-specific and discount some physical activity (e.g., domestic tasks) thus further limiting their use [6]. Functional assessments provide a measure of performance at the time of assessment; however, this assessment may not reflect the individual’s performance throughout the day [3]. Additionally, little is known about the validity and reliability of self-report for assessing sedentary behavior [8] and physical activity [9] in older adults. Recalling physical activity is a complex cognitive task. Self-report of physical activity is likely to be difficult for those with memory limitations [10]. With the insufficiency of these various approaches in providing comprehensive activity data, the development and use of lightweight, sensitive, activity monitors has increased in population-based research [11].

One such device, the activPAL, has been shown to be valid and reliable in children [12,13] and adult populations [14] in relation to determining body posture, transitions between body posture positions, normal stepping and cadence. It has also been shown to be valid in measuring stepping and cadence in community-dwelling older adults [15], valid in classifying body posture positions and transitions among in-patient and healthy older adults [16] and feasible for use with older adults in residential care homes [17]. It uses algorithms to interpret an individual’s physical activity and posture in relation to time [3]. The activPAL is lightweight, does not require cabling, is smaller than other available devices and provides step count and accelerometry data in an accessible format, all rendering it suitable for research [18]. This small device, designed to be worn midway on the thigh, allows older adults to continue with their normal activities during data collection. A small, lightweight device without cabling may also reduce the risk of skin tears or pressure wounds for the wearer.

The aim of this rapid review was to summarize and assess the quality of current observational literature measuring physical activity and sedentary behavior in older adults using the activPAL. A rapid review method was selected rather than a systematic review so that evidence was synthesized in a timely and less resource-intensive manner [19]. To our knowledge, no other such review of the activPAL exists, however, an earlier systematic review examined multiple accelerometer-based body-worn sensors (including the activPAL) in studies measuring physical activity of older adults [6]. In that review, the most recent article was published in 2011, no quality assessment was completed, the activPAL was not studied in detail and its limitations were not discussed. Therefore, this rapid review fills this gap by providing a summary and assessment of current literature on the ActivPAL.

## 2. Methods

### 2.1. Search Strategy

A health sciences librarian developed a search for articles using a Discovery Service of databases including MEDLINE, CINAHL and SPORTDiscus. The search entries: activpal* AND (elder* or older w2 (adult* or people or person* or men or women) or seniors or geriatric* or gerontology* or “old age” or SU (aged) were used. The search also included a bibliography of literature compiled by the activPAL creators.

After non-peer-reviewed articles and duplicates were removed, the first author (CC) screened articles by reading the titles and abstracts. For those articles selected for full review, data were extracted by one reviewer (CC).

### 2.2. Inclusion and Exclusion Criteria

No limit was placed on an initial date but articles were searched up to and including 6 July 2015. Inclusion criteria were: (1) the target population included participants with sample mean age of 65 years or older; (2) activPAL was used to observe activity or sedentary behavior; and (3) at least one of the following activPAL outcomes was reported: step count, hours upright, hours sitting/lying, hours stepping, or hours standing. The search was limited to English-language articles from the peer-reviewed literature with an observational design. Methodological papers, case reports and experimental studies (i.e., randomized controlled trials) were excluded as we were interested only in those studies whose primary purpose was using the ActivPAL to describe the patterns of physical activity and sedentary behavior. Both cross-sectional and cohort studies provided information on the daily physical activity and sedentary behavior patterns in older adults. Experimental studies examining the effect of interventions on physical activity and validation studies examining activity patterns in controlled settings were excluded, as they did not provide information on older adult’s usual patterns of activity and sedentary behavior.

### 2.3. Assessment of Methodological Quality

To assess the quality of the articles included in the review, a modified Quality Assessment and Validity Tool (QAVT) was used [20]. This tool was developed by Estabrooks et al. [21] and was the most appropriate tool to assess the observational studies extracted in this review. Four domains are assessed in the instrument: design of the study, sample characteristics, measurement and statistical analysis. Total scores of 0–4 indicate low overall quality, total scores of 5–9 indicate medium quality and total scores of 10–14 indicate high quality.

Before a full quality assessment took place, three raters (CC, PS, CI) discussed each scale to ensure that their interpretations of the questions and criteria were the same. Each rater then independently scored two articles before coming to a consensus on any discrepancies in the interpretation of the scales and scores. For the two articles rated initially, percent agreements among the 3 raters (CC, PS and CI) were 73% for individual items, 63% for each domain and 100% for overall study rating. Pairs of reviewers (CC, PS, CI) independently assessed each of the 22 articles and discussed any discrepancies. One reviewer (CC) reviewed all articles. PS reviewed 12 articles and CI reviewed the remaining 10 articles. Cohen’s Kappa was calculated to determine interrater reliability [22]. Interrater reliabilities for individual items were Kappa = 0.649 (*p* < 0.001) and Kappa = 0.445 (*p* < 0.001) for each of the domains. Following separate reviews by each rater, the two raters met and compared each scored item. Any divergent items were discussed and a consensus was reached.

## 3. Results

### 3.1. Study Selection

**Figure 1:**
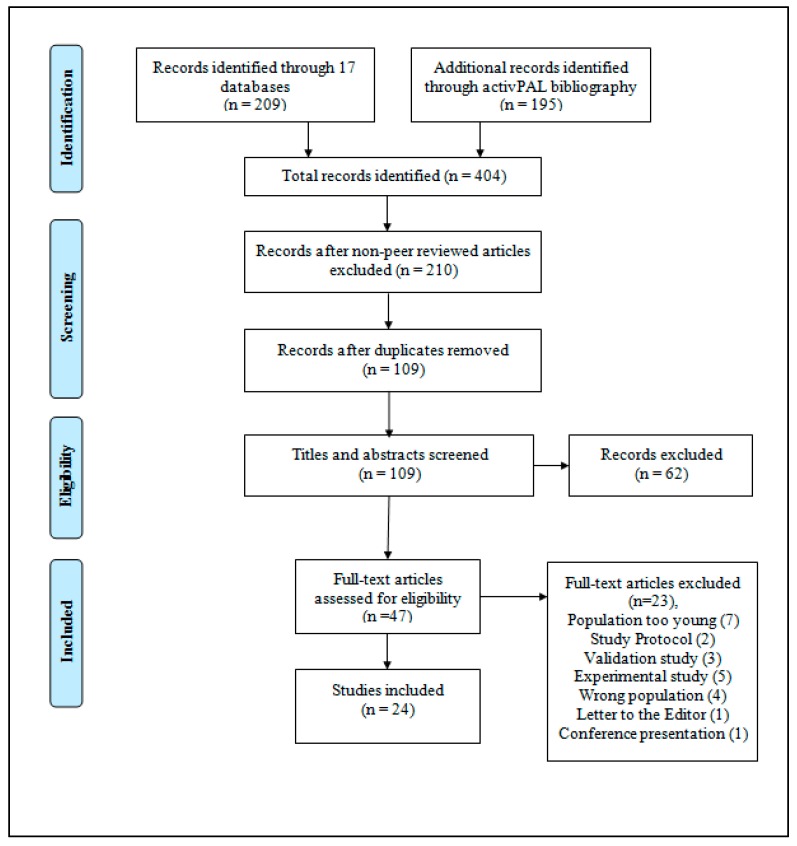
Study selection according to PRISMA guidelines.

Of the 404 articles retrieved, 209 articles were retrieved from the database search and 195 from the activPAL bibliography, 194 articles did not meet the criteria which left 210 articles. After removal of duplicates, 109 articles remained. These titles and abstracts were screened leaving 47 articles for full-text review. After full-text review, a further 23 articles were excluded leaving 24 for inclusion (Figure 1).

### 3.2. Study Characteristics

Included articles were published between 2007 and 2015 and originated from the United Kingdom (*n* = 13), Australia (*n* = 6), Germany (*n* = 3), Canada (*n* = 1) and USA (*n* = 1). The majority of the articles (*n* = 21) were cross-sectional studies while those remaining were prospective longitudinal cohort studies (*n* = 3). Of the 24 studies, one examined older adults from residential aged care [17], six focused on older hospital inpatients [3,18,23,24,25,26] eight examined community-dwelling older adults [8,27,28,29,30,31,32,33] and nine focused on individuals from outpatient clinics [7,34,35,36,37,38,39,40,41]. Study sample sizes ranged from 10 to 1324 people.

### 3.3. Participant Characteristics

The mean age of participants in the studies ranged from 66.0 to 84.2 years of age. Older adults from outpatient clinics were diagnosed with cancer (two samples), intermittent claudication (two samples), Parkinson’s disease (two samples), leg ulcers (one sample) or stroke (two samples) (Table 1).

### 3.4. Physical Activity Outcomes

The activPAL was worn for 24 h per day over seven days in 71% (*n* = 17) of the studies [7,8,17,26,27,28,29,30,33,34,35,36,37,38,39,40,41]. Others reported a wear time of six to seven hours in one day [18], 24 h per day over one to two [23,25], three, or eight days [3,24] and waking hours over three or five days [31,32]. Average daily step count, daily hours stepping, daily hours upright and daily hours sitting/lying were the most commonly reported variables although there was considerable variability in reporting between studies, as below.

Twelve studies reported mean or median step count per day [7,17,24,26,27,34,35,36,37,39,40,41]. The lowest step count was found in a sample of older adults post-opeeratively following hip fracture with a median of 35.7 (standard deviation [SD] = 35.7) steps per day [26]. The highest step count was found in a control group of healthy Scottish community-dwelling older adults with a mean of 8864 steps per day (SD = 3110) [36].

Ten studies reported mean or median hours stepping per day [17,23,24,28,29,32,35,38,39,41]. The smallest amount of time spent stepping was seen in 54 older adults following a hip fracture with a median of 0.13 (interquartile range = 0.05–0.27) h per day [24]. Community-dwelling older adults from a UK sample had the highest hours of stepping with a mean of 5.02 (SD = 1.67) h [41]. Four articles represented time stepping as a percentage of total time [7,26,30] or as a percentage of waking hours [18].

Mean or median hours spent standing per day was reported in five articles [17,23,25,35,39] with the minimum reported as 0.8 (SD = 1.27) h per day for individuals in palliative care [23]. The maximum mean daily hours spent standing was 3.4 (SD = 1.6) h in a sample of individuals with lung or upper gastrointestinal cancer [35]. Two articles reported standing data as a percentage of waking time [18,26].

### 3.5. Sedentary Behavior Outcomes

Five articles reported mean daily hours upright with a range of 1.17 (SD = 0.84) h to 4.3 (SD = 2.0) h [3,24,27,31,37]. Daily hours upright were calculated by combining the time spent standing and stepping. The lowest mean daily hours spent upright was found in adult inpatients with a lower limb orthopaedic condition [24] while the highest mean time was found in individuals with thoracic cancer [37]. One article reported upright data as a percentage of time with the activPAL in place [34].

Ten studies reported daily mean hours sitting/lying [8,17,23,24,25,27,32,35,37,39] with the minimum mean hours spent sitting/lying as 8.2 (SD = 2.0) in an Australian sample of 23 healthy controls [39]. The maximum mean daily hours spent sitting/lying was 23.0 (SD = 0.7) in adult inpatients with a lower limb orthopaedic condition [24]. Some articles reported daily mean hours including sleep time [25] while others did not [8]. Three articles reported time spent sitting/lying as a percentage either of total monitored time per day [26,30,34] or of daily waking hours [18].

### 3.6. Limitations of the activPAL and Reporting Limitations

Seven articles briefly discuss limitations of activPAL data classification [3,8,17,25,31,33,40]. The activPAL was found to inaccurately classify lower limb movement as steps due to hesitancy, shuffling and pausing in frail elderly hospital patients [3]. Others hypothesize that the ability to detect steps decreases at very slow walking speeds and the activPAL can underreport step count [17,32,40], specifically, at gait speeds ≤0.47 m/s [16]. With inpatient and community-dwelling samples, the activPAL was found to misclassify postures such as irregular sitting styles [25]. One study noted that the activPAL could not distinguish between sitting/lying, a source of estimation error [8]. All seven samples had a mean age range of 71.1–84.2 years of age. This age range was older than the age range of the 17 other articles that reported no discrepancy in classification of data by the activPAL (mean age range of 66–79.9 years).

There was considerable variability in the reporting of activPAL outcomes. For example, among those who documented sedentary behavior, some reported percentage of time spent sitting/lying [18,26,30,34], some reported total hours sitting/lying [23,24,25,37], while some reported waking hours sitting/lying [8,17,27,32,35,39]. Among those who documented physical activity, some studies reported time spent stepping only [18,23,28,29,30,33,38] while others reported step count only [27,34,36,37,40].

### 3.7. Quality Assessment

Of the 24 observational studies, seven were of low quality [23,31,32,34,37,38,41] while the remaining 17 were of medium quality. Most of the observational studies lacked probability sampling, the use of a theoretical model/framework, the use of a scale with an internal consistency >0.70 and the management of outliers. Several observational studies did not mention justification of sample size and protection of anonymity. Domain and overall quality scores are reported in Table 2.

## 4. Discussion

This rapid review provides a brief exploration of the direction and quality of existing literature on the use of the activPAL to track physical activity and sedentary behaviors. Most of the studies were performed in the UK where the activPAL was created. Eleven of the studies in this review examined both physical activity and sedentary behavior patterns of the participants [17,18,23,24,26,27,30,34,35,37,39] while eight examined physical activity patterns alone [7,28,29,33,36,38,40,41] and five examined sedentary behavior only [3,8,25,31,32]. While most studies looked at activity levels and sedentary behavior in the community, only one study examined activity and sedentary behavior in long-term care [17].

There is a lack of consistency in the reporting of physical activity and sedentary behavior outcome variables even when the same device was used across studies. Comparison across several studies or meta-analysis was therefore not warranted. A systematic review of multiple accelerometer-based body-worn sensors in older adults found similar limitations in reported data [6]. A systematic review of the commonly-used Actigraph noted similar findings [42]. There are few articles that provide specific guidelines for standardizing accelerometer data [43,44] and none to our knowledge specifically for the activPAL. The lack of standardized guidelines for reporting physical activity and sedentary behavior outcomes highlights the need for more research or the development of a consensus on best practice recommendations.

No studies were assessed as high quality. Several observational studies scored lowest in the measurement category, the use of a theoretical model/framework and the use of a scale with an internal consistency >0.70 were the most common deficiencies. These may relate to a lack of reporting rather than conduct but we were unable to identify if this was the case. Methods by which anonymity was protected or sample size was justified were underreported although this was noticeably difficult for studies that included small samples such as individuals with Chronic Obstructive Pulmonary Disease in a hospital [25].

Many of the articles describing the limitations of the activPAL included older (71+ years) participants. The activPAL may not appropriately capture certain types of physical activity or sedentary behavior in a frail, elderly population which is more likely to have a slower walking speed, may use walking aids and may spend time in chairs with a relatively high angle of recline (i.e., recliner chairs). In an exploratory study, activPAL misclassified as standing, residents who sat on elevated recliner chairs [45]. Limitations of the activPAL need to be considered in future studies or in clinical application.

This study has several limitations. The small scope and number of studies limits generalizability. The use of a rapid review, rather than a systematic review, to streamline the review process, including having a single reviewer, may have introduced sampling or selection bias into our search. Decisions regarding article inclusion or exclusion could have been influenced by the reviewer’s prior knowledge or understanding of the content area. Further, a methodological limitation of the quality assessment is the moderate Kappa scores.

Nevertheless, this rapid review provides an overview of the quality and direction of existing literature. The relatively small number of articles revealed by this rapid review may be due to the success of other accelerometers in profiling the physical activity or sedentary behavior of older adults. Of the 134 studies included in the Taralsden et al. Systematic Review, the majority of the activity trackers were Actigraph/MTI/ActiWatch/Mini-Motion logger (34 studies), while one study was found for other accelerometers including activPAL, Dynaport, Actical, Dynalog, Actilog and Lifecorder [6].

## 5. Conclusions

This rapid review demonstrates a lack of high quality articles using the activPAL as the objective monitoring device of choice, particularly in older adults residing in long-term care. The activPAL may be a feasible monitoring device in the older adult population but more investigations are needed to examine its use in frail older adults. More detailed guidelines for reporting activity monitor outcomes are needed to ensure reporting consistency and adherence to guidelines across studies.

## Figures and Tables

**Table 1:** Study characteristics.

Author	Country	Study Design	Population and Setting	*n*	Mean Age, Years (Standard Deviation)	Time Worn (h)	Activity Outcome Measured Daily Average (SD)
Clarke-Moloney et al., 2007 [34]	UK	Cross-sectional	Patients with leg ulcers and healthy age-matched controls	50 (24 M, 26 F)	70.5 (median) (IQR–NR)	24 h × 7 days	LU median (range) % time upright = 30.8 (8.4–30.6) C median (range) % time upright = 27.7 (17.7–42.6) LU median (range) % time spent sitting/lying = 69.2 (49.4–91.6) C median (range) % time spent sitting/lying = 72.3 (57.4–82.3) LU median (range) step count = 6685 (2074–17,999) C median (range) step count = 8750 (4917–16,043)
Godfrey et al., 2010 [23]	UK	Cross-sectional	Palliative care patients	40 (23 M, 17 F)	68.4 (11.9)	24 h × 1 day	Hours sitting/lying = 22.6 (2.12) Hours standing = 0.8 (1.27) Hours stepping = 0.21 (0.47)
Grant et al., 2010 [3]	UK	Cross-sectional	Older adults from two hospitals	70 (25 M, 45 F)	City: 81.8 (6.7) Rural: 79.4 (4.7) Hospital: 74.7 (7.9) Healthy: 73.7 (5.5)	24 h × 8 days	City ward hours upright = 1.17 (0.84) Rural ward hours upright = 1.34 (0.69) Day hospital hours upright = 3.89 (1.88) Community hospital hours upright = 6.01 (1.87)
Maddocks et al., 2010 [35]	UK	Cross-sectional	Individuals with lung or upper gastrointestinal cancer from an oncology clinic	60 (40 M, 20 F)	68.0 (9.0)	24 h × 7 days	Waking hours sitting/lying = 10.8 (2.5) Hours standing = 3.4 (1.6) Hours stepping = 1.0 (0.7) Step count = 4244 (2939)
Lord et al., 2011 [27]	UK	Cross-sectional	Community-dwelling older adults	56 (26 M, 30 F)	78.9 (4.9)	24 h × 7 days	Hours upright = 4.18 (1.73) Waking hours sitting/lying = 12.46 (1.94) Step count = 6343 (2807)
Clarke et al., 2012 [36]	UK	Cross-sectional	Individuals with IC from an outpatient clinic and healthy age-matched controls	60 (36 M, 24 F)	IC: 67.2 (9.7) C: 66.8 (10.5)	24 h × 7 days	C group step count = 8864 (3110) IC group step count = 6524 (2710)
Klenk et al., 2012 [28]	Germany	Cross-sectional	Community-dwelling older adults	1324 (747 M, 577 F)	74.6 (NR)	24 h × 7 days	Men hours stepping = 1.74 (0.85) Women hours stepping = 1.72 (0.80)
Klenk et al., 2012 [29]	Germany	Cross-sectional	Community-dwelling older adults	1253 (710 M, 543 F)	75.6 (6.5)	24 h × 7 days	Men hours stepping = 1.75 (0.69) Women hours stepping = 1.72 (0.66)
Maddocks et al., 2012 [37]	UK	Cross-sectional	Individuals with thoracic cancer from an outpatient clinic	84 (54 M, 30 F)	66.0 (9.0)	24 h × 7 days	Total hours sitting/lying = 19.7 (2.1) Hours upright = 4.3 (2.0) Step count = 4246 (2983)
Lord et al., 2013 [7]	UK	Cross-sectional	Patients with newly diagnosed PD from secondary care services and healthy controls	186 (112 M, 74 F)	PD: 67.3 (9.9) C: 69.2 (7.7)	24 h × 7 days	C group % total time stepping = 6.9 (2.3) PD group % total time stepping = 5.1 (2) C group step count = 7816 (5452) PD group step count = 5452 (2501)
Peiris et al., 2013 [24]	Australia	Cross-sectional	Adult inpatients with lower limb orthopaedic condition	54 (34 M, 40 F)	74.0 (11.0)	24 h × 3 days	Median (IQR) step count = 398 (140–993) Median (IQR) hours stepping = 0.13 (0.05–0.27) Hours upright = 0.97 (0.62) Hours sitting/lying = 23.0 (0.7)
Reid et al., 2013 [17]	Australia	Cross-sectional	Residential aged care residents	31 (11 M, 20 F)	84.2 (NR)	24 h × 7 days	Waking hours sitting/lying = 12.4 (IQR = 1.7) Hours standing = 1.9 (IQR = 1.3) Hours stepping = 0.36 (IQR = 0.40) Step count = 1055 (IQR = 1110)
Rowlands et al., 2014 [25]	Australia	Cross-sectional	Individuals with COPD in a hospital	10 (4 M, 6 F)	75.9 (9.7)	24 h × 1–2 days	Total hours sitting/lying = 22.15 (1.33) Total hours standing = 1.84 (1.34)
Aguilar-Farías et al., 2014 [8]	Australia	Cross-sectional	Community-dwelling older adults	41 (14 M, 27 F)	74.5 (7.6)	24 h × 7 days	Waking hours sitting/lying = 9.60 (1.66) Waking weekday hours sitting/lying = 9.55 (1.64) Waking weekend hours sitting/lying = 9.68 (1.96)
Godfrey et al., 2014 [30]	UK	Cross-sectional	Community-dwelling older adults	98 (50 M, 48 F)	69.1 (7.6)	24 h × 7 days	Employed % time sitting/lying = 78.00 (6.17) Employed % time stepping = 6.24 (2.18) Retired % time sitting/lying = 74.73 (5.77) Retired % time stepping = 1.76 (0.15)
Mactier et al., 2014 [38]	UK	Cross-sectional	Individuals newly diagnosed with PD	111 (77 M, 34 F)	68.7 (median) (IQR—60.9–75.0)	24 h × 7 days	No falls median (IQR) % time stepping = 5.1 (3.9–6.5) Single fall median (IQR) % time stepping = 4.9 (3.4–5.7) Recurrent falls median (IQR) % time stepping = 5.1 (3.9–6.2)
Salbach et al., 2014 [31]	Canada	Cross-sectional	Community-dwelling PS	16 (14 M, 2 F)	71.1 (9.7)	Waking hours × 5 days	Hours upright = 3.15 (2.27)
Davenport et al., 2015 [26]	Australia	Cross-sectional	Older adults post-surgical from hip fractures	20 (2 M, 18 F)	79.1 (9.3)	24 h × 7 days	% of day sitting/lying = 98.9 (1.0) % of day standing = 1.1(1.0) % of day stepping = 0.05(0.09) Step Count = 35.7 (80.4)
Duncan et al., 2015 [40]	UK	Prospective cohort	Individuals with acute stroke admitted to hospital or an outpatient clinic	84 (56 M, 28 F)	72.3 (median) (IQR—65.2–80.5)	24 h × 7 days	Median (IQR) step count in thousands at 1 month = 2.841 (1.419–5.723) Median (IQR) step count in thousands at 6 months = 4.047 (2.056–5.822) Median (IQR) step count in thousands at 12 months = 4.314 (1.657–6.890)
English et al., 2015 [39]	Australia	Cross-sectional	Individual post-stroke and healthy controls	63 (41 M, 22 F)	68.4 (10.0)	24 h × 7 days	PS hours sitting = 10.9 (2.0) C hours sitting = 8.2 (2.0) PS hours standing = 2.6 (1.5) C hours standing = 5.2 (1.7) PS hours stepping = 1.1 (0.8) C hours stepping = 2.2(0.8) PS step count = 2411 (1835) C step count = 5314 (2100)
Gennuso et al., 2015 [32]	USA	Cross-sectional	Community-dwelling older adults	44 (16 M, 28 F)	70.0 (8.0)	Waking hours × 3 days	Median (25–75%) M hours sitting = 9.6 (8.7–11.1) Median (25–75%) F hours sitting = 9.3 (7.9–10.3)
Klenk et al., 2015 [33]	Germany	Prospective cohort	Community-dwelling older adults	1214 (693 M, 521 F)	75.6 (6.5)	24 h × 7 days	Hours stepping = 1.73 (0.67)
Kunkel et al., 2015 [18]	UK	Prospective cohort	Individuals post-stroke in a hospital	76 (39 M, 35 F)	76.0 (11.0)	6–7 h × 1 day	PS % waking time sitting/lying = 94 PS % waking time standing = 4 PS % waking time stepping = 2
Stansfield et al., 2015 [41]	UK	Cross-sectional	Individuals with IC from an outpatient service and healthy controls	60 (36 M, 24 F)	IC: 67.2 (9.7) C: 66.8 (10.5)	24 h × 7 days	C group hours stepping = 5.00 (1.17) C group step count = 8692 (2945) IC group hours stepping = 5.02 (1.67) IC group step count = 6531 (2712)

Abbreviations: C = Control group, COPD = Chronic Obstructive Pulmonary Disease, F = Female, IC = Intermittent Claudication, IQR = Interquartile range, M = Male, *n* = Sample size, NR = Not Reported, PD = Parkinson’s Disease, PS = Individuals post-stroke, UK = United Kingdom, LU = Patients with leg ulcers.

**Table 2:** Quality assessment domain and overall ratings for observational studies.

Study Reference	Design (/2)	Sample (/4)	Measurement (/6)	Statistical Analysis (/2)	Overall Rating (/14)
Clarke-Moloney et al., 2007 [34]	1	1	0	1	3 (LOW)
Godfrey et al., 2010 [23]	1	1	1	0	3 (LOW)
Grant et al., 2010 [3]	1	1	3	1	6 (MEDIUM)
Maddocks et al., 2010 [35]	1	3	3	0	7 (MEDIUM)
Lord et al., 2011 [27]	1	1	4	1	7 (MEDIUM)
Clarke et al., 2012 [36]	1	1	3	0	5 (MEDIUM)
Klenk et al., 2012 [28]	2	2	3	1	8 (MEDIUM)
Klenk et al., 2012 [29]	2	2	1	0	5 (MEDIUM)
Maddocks et al., 2012 [37]	1	2	1	0	4 (LOW)
Lord et al., 2013 [7]	1	1	2	1	5 (MEDIUM)
Peiris et al., 2013 [24]	2	1	3	0	6 (MEDIUM)
Reid et al., 2013 [17]	2	2	3	0	7 (MEDIUM)
Rowlands et al., 2014 [25]	1	1	3	0	5 (MEDIUM)
Aguilar-Farías et al., 2014 [8]	1	1	3	2	7 (MEDIUM)
Godfrey et al., 2014 [30]	1	1	3	0	5 (MEDIUM)
Mactier et al., 2014 [38]	2	1	1	0	4 (LOW)
Salbach et al., 2014 [31]	1	0	1	2	4 (LOW)
Davenport et al., 2015 [26]	2	2	3	0	7 (MEDIUM)
Duncan et al., 2015 [40]	1	2	3	2	8 (MEDIUM)
English et al., 2015 [39]	1	1	2	1	5 (MEDIUM)
Gennuso et al., 2015 [32]	1	1	1	0	3 (LOW)
Klenk et al., 2015 [33]	2	2	3	0	7 (MEDIUM)
Kunkel et al., 2015 [18]	1	1	3	1	6 (MEDIUM)
Stansfield et al., 2015 [41]	1	0	3	0	4 (LOW)
